# Ocular gene transfer in the spotlight: implications of newspaper content for clinical communications

**DOI:** 10.1186/1472-6939-15-58

**Published:** 2014-07-16

**Authors:** Shelly Benjaminy, Tania Bubela

**Affiliations:** 1 Department of Public Health Sciences, School of Public Health, Edmonton Clinic Health Academy, University of Alberta, 11405 87 Ave NW, Edmonton, Alberta, Canada

**Keywords:** Gene transfer, Gene therapy, Media, Newspaper coverage, Therapeutic misconception, Ethics, Informed consent

## Abstract

**Background:**

Ocular gene transfer clinical trials are raising hopes for blindness treatments and attracting media attention. News media provide an accessible health information source for patients and the public, but are often criticized for overemphasizing benefits and underplaying risks of novel biomedical interventions. Overly optimistic portrayals of unproven interventions may influence public and patient expectations; the latter may cause patients to downplay risks and over-emphasize benefits, with implications for informed consent for clinical trials. We analyze the news media communications landscape about ocular gene transfer and make recommendations for improving communications between clinicians and potential trial participants in light of media coverage.

**Methods:**

We analyzed leading newspaper articles about ocular gene transfer (1990-2012) from United States (n = 55), Canada (n = 26), and United Kingdom (n = 77) from Factiva and Canadian Newsstand databases using pre-defined coding categories. We evaluated the content of newspaper articles about ocular gene transfer for hereditary retinopathies, exploring representations of framing techniques, research design, risks/benefits, and translational timelines.

**Results:**

The dominant frame in 61% of stories was a celebration of progress, followed by human-interest in 30% of stories. Missing from the positive frames were explanations of research design; articles conflated clinical research with treatment. Conflicts-of-interest and funding sources were similarly omitted. Attention was directed to the benefits of gene transfer, while risks were only reported in 43% of articles. A range of visual outcomes was described from slowing vision loss to cure, but the latter was the most frequently represented even though it is clinically infeasible. Despite the prominence of visual benefit portrayals, 87% of the articles failed to provide timelines for the commencement of clinical trials or for clinical implementation.

**Conclusions:**

Our analysis confirms that despite many initiatives to improve media communications about experimental biotechnologies, media coverage remains overly optimistic and omits important information. In light of these findings, our recommendations focus on the need for clinicians account for media coverage in their communications with patients, especially in the context of clinical trial enrolment. The development of evidence-based communication strategies will facilitate informed consent and promote the ethical translation of this biotechnology.

## Background

The field of gene transfer, colloquially known as ‘gene therapy’ , has followed a trajectory of high hopes and high profile failures. Historical abuses, including non-disclosure of serious adverse events and highly publicized conflicts-of-interest, have damaged its reputation [1-3], eroded public trust, and presented significant setbacks for its clinical development [4,5].

Ocular applications may yet rescue the reputation of gene transfer with successful clinical trials beginning in 2007. Phase I clinical trials for Leber congenital amaurosis (LCA), a rare, blinding retinopathy, established both indexes of safety and improved visual function, despite the small size and safety focus of first-in-human phase I clinical trials [6-8]. Phase I trials are followed by larger phase II trials that continue to test safety and establish preliminary measures of efficacy. Phase III trials, most commonly randomized, controlled trials, are then statistically powered to test efficacy for primary (e.g., visual acuity) and secondary (e.g., retinal imaging) outcome measures. Sustained safety results for LCA [9] have led to a phase III clinical trial at the University of Pennsylvania, which is currently recruiting children (clinicaltrials.gov: NCT00999609). Developments in LCA trials have served as an impetus for further clinical trials for related ocular diseases [10], including Stargardt disease (clinicaltrials.gov: NCT01367444), retinitis pigmentosa (clinicaltrials.gov: NCT01482195), Usher syndrome (clinicaltrials.gov: NCT01505062), and choroideremia (clinicaltrials.gov: NCT01461213).

These recent advances in ocular gene transfer have triggered media attention [11], raising patient hopes [12] for the mediation of previously untreatable genetic diseases. News media are the most accessible source of information on biomedical research for the public and patients [13]. Media coverage, therefore, if exaggerated or misrepresentative of gene transfer, may influence societal and patient expectations.

News media are often blamed for distorting the impact of biotechnology research through overly aversive or favorable communications [13,14]. Media use frames (simplified, interpretive representations of an issue) to highlight the importance of an issue and how it should be addressed [15]. Frames often bias contentious scientific issues by placing greater emphasis on certain considerations while omitting others [16]. For example human embryonic stem cell research may be framed as ethically unjustifiable for its destruction of embryos or as an ethical imperative to provide cures for otherwise incurable diseases [17,18]. Biomedical reporting is often criticized for its framing that uncritically celebrates progress in research [19]. Such framing emphasizes benefits over risks [20], and omits crucial information such as funding sources and potential conflicts-of-interest [21,22]. The coverage often presents pre-clinical research (i.e., in-vitro or animal studies) as an imminent therapy or cure [20].

From a clinical perspective, the uncritical progress frame may lead clinicians, patients, and their families to form high expectations, only to be disappointed when reality falls short of hope, causing disillusionment among clinicians and despair among patient communities [23]. In the context of clinical trials, overly positive media coverage about research in progress may present challenges to enrolment and informed consent in ongoing or subsequent clinical trials by heightening patient expectations for therapeutic benefits [24]. In particular, potential participants may misunderstand the safety focus of early-phase trials and expect therapeutic outcomes [25].

As ocular gene transfer clinical trials for hereditary retinopathies increase in number and reach phase III, attention must be directed at facilitating ethical communications of risks and potential benefits for recruitment of clinical trial participants. Such communications will occur in an environment of heightened media coverage, with media influencing patient and public perspectives about pre-clinical and clinical gene transfer research. Here, we ask how newspaper articles report on ocular gene transfer for hereditary retinopathies, exploring errors of omission and biases in representations of framing, risks and benefits, as well as therapeutic potential. Our analysis suggests the need for clinicians to account for media coverage in their communications with patients, especially in the context of clinical trial enrolment. Evidence-informed communication strategies set against the media landscape will ensure the ethical translation of this biotechnology as it moves forward to clinical application.

## Methods

We examined newspaper communication about ocular gene transfer between January 1, 1990 and June 30, 2012 in the top 50 United States (US), Canadian, and United Kingdom (UK) newspapers by circulation [26]. We selected 1990 as our start year because it corresponds to the first approved gene transfer clinical trial. These countries ranked within the top ten for production of gene transfer trials [27]; ocular gene transfer trials have commenced in the US and UK and will shortly commence in Canada. We used search algorithms for ‘ocular gene transfer’ in media databases Factiva and Canadian Newsstand. The initial search produced 2070 articles (84 Canadian, 647 UK, and 1339 US). After reading each article, we excluded all but original articles about gene transfer targeting retinopathies, resulting in 158 articles (26 Canadian, 55 US, and 77 UK) for analysis (Table 1).

**Table 1:** Search algorithms for ocular gene transfer newspaper articles in Factiva and Canadian Newsstand

**Country**	**Search strategy**^**a**^	**Number of articles**	**Number of articles on retinopathies for analysis**
United States	At least one of these words: blind* ocular ophtha* vision sight retin* eye	1339	55
	This exact phrase: gene therapy		
Canada	TITLE (blind* OR ocular OR ophtha* OR vision OR sight OR retin* OR eye) AND (gene therap*)	647	26
United Kingdom	At least one of these words: blind* ocular ophtha* vision sight retin* eye	84	77
	This exact phrase: gene therapy		

^a^A truncation symbol (*) was used to capture alternative word endings of search terms (e.g. retin* captures retina, retinas, retinopathy, retinopathies, etc.).

We used pre-determined coding categories for our content analysis. The coding categories were modified from pre-existing studies about media coverage of health research and biotechnologies [28,29] and adapted to include issues raised in similar studies [30-32]. Furthermore, the coding categories were developed in response to issues raised by forty-one semi-structured interviews with stakeholders of a phase I ocular gene transfer clinical trial (choroideremia patients, clinicians, and patient advocates). A thematic analysis of these interviews revealed high therapeutic hopes, limited attention to risks, and confusion about the timelines for the clinical application of gene transfer among patients [33]. The final coding categories investigated the attention structure of newspaper articles (word count, page number, news format), disease representations, key categories of spokespeople, one dominant frame for each article, use of human-interest stories, tone of coverage, description of methods, representations of timelines for translational research and clinical implementation, and representations of risks/benefits. Tone of coverage was determined using a combination of quantitative (number of benefits stated versus number of risks) and qualitative (prominence and stated magnitude of benefits versus risks) assessments. If the article stated more benefits than risks, its tone was positive. However, where benefits and risks were equal in number, we considered their respective magnitude. For example, a benefit of “cure for blindness” versus the economic risk of “expense of the clinical trial” also indicated a positive tone.

A research assistant coded all articles, and one of the authors [SB] independently coded 19% to verify that a second coder would arrive at the same conclusion as the main coder. We calculated Cohen’s Kappa for inter-coder reliability (SPSS 18: IBM). Kappa scores ranged between 0.71-1.0, displaying acceptable inter-coder agreement [34]. To accommodate the small sample size of Canadian newspaper articles, we used Fischer’s chi squared tests (STATA 11: StataCorp 2009) to assess differences in coverage between countries. We used a Kruskal-Wallis Analysis of Variance (STATA 11: StataCorp 2009)—a non-parametric alternative to a one-way analysis of variance—to assess differences in the median number of benefit and risk representations between countries. We combined articles from the three countries when there was no significant difference in coverage (α = 0.05).

## Results

**Figure 1:**
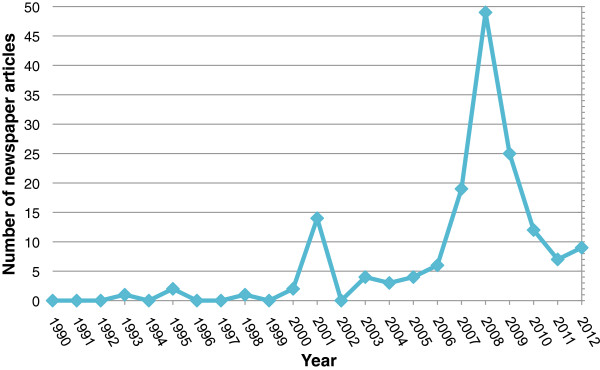
Distribution of newspaper articles about ocular gene transfer over time.

Most articles were published in the main news section with only 1% of articles appearing as front-page news. The average article word length was 615 words. Ninety two percent of articles were formatted as “latest news”, and a minority was formatted as interviews (3%), commentaries (3%), and editorials (2%). The articles covered a variety of retinopathies; some currently under the investigation of clinical trials and some that have not advanced beyond pre-clinical studies (Table 2). The distribution of newspaper articles over time is displayed in Figure 1.

**Table 2:** Retinopathies represented in newspaper articles compared to phase of clinical trials registered in clinicaltrials.gov

**Disease**^**b**^**(number of articles**^**a**^**)**	**Nature of retinopathy**	**Clinical trial number**	**Clinical trial phase**	**Gene targeted**	**Vector type**	**Number (n) and age of participants**
Leber congential amaurosis (84)	Progressive retinopathy with severe visual problems beginning in infancy.	NCT00999609	III	RPE65	Adeno-associated viral vector; AAV2-hRPE65v2	n = 24; 3 years and older
		NCT00749957	I/II	RPE65	Recombinant adeno-associated viral vector; rAAV2-CB-hRPE65	n = 12; 6 years and older
		NCT01208389	I/II	RPE65	Adeno-associated viral vector; AAV2-hRPE65v2	n = 12; 8 years and older
		NCT00643747	I/II	RPE65	Recombinant adeno-associated viral vector; rAAV 2/2.hRPE65p.hRPE65	n = 12; 5-30 years old
		NCT01496040	I/II	RPE65	Recombinant adeno-associated viral vector; rAAV-2/4.hRPE65	n = 9; 6-50 years old
		NCT00481546	I	RPE65	Recombinant adeno-associated viral vector; rAAV2-CBSB-hRPE65	n = 15; 8 years and older
		NCT00821340	I	RPE65	Recombinant adeno-associated viral vector; rAAV2-hRPE65	n = 10; 8 years and older
		NCT00516477	I	RPE65	Adeno-associated viral vector; AAV2-hRPE65v2	n = 12; 8 years and older
Retinitis pigmentosa (37)	Progressive retinopathy characterized by gradual peripheral vision loss	NCT01482195	I	MERTK	Recombinant adeno-associated viral vector; rAAV2-VMD2-hMERTK	n = 6; 14-70 years old
Age-related macular degeneration (24)	Progressive central vision loss usually occurring in older adults	NCT01301443	I	Genes that encode endostatin and angiostatin	Lentiviral vector; RetinoStat	n = 18; 50 years and older
		NCT01678872	I	Genes that encode endostatin and angiostatin	Lentiviral vector; RetinoStat	n = 18; 50 years and older
Choroideremia (7)	Progressive retinopathy characterized by gradual peripheral vision loss	NCT01461213	I/II	REP1	Adeno-associated viral vector; AAV.REP1	n = 12; 18 years and older
Stargardt macular degeneration (7)	Progressive degeneration of the macula causing central vision loss beginning in childhood	NCT01367444	I/IIa	ABCA4	Lentiviral vector; StarGen	n = 28; 18 years and older
Usher syndrome (7)	Progressive retinopathy (retinitis pigmentosa) combined with hearing loss	NCT01505062	I/IIa	MYO7A	Lentiviral vector; UshStat	n = 18; 18 years and older

^a^Some articles targeted more than a single retinopathy.

^b^Note that 18 articles referred to gene transfer targeting color blindness, one referred to retinocshisis, and one referred to congenital stationary night blindness, even though no clinical trials for these conditions were registered in clinicaltrials.gov.

### Dominant spokespeople and frames

The most frequently quoted spokespeople were researchers from universities, research institutes or government; followed by patient advocacy organization representatives and affected individuals and their families (Table 3). The majority of Canadian and UK articles quoted a researcher compared to 38% of US articles (Table 3). Industry quotes were scarce in all countries with only a single article interviewing a private sector scientist and a second representing the views of a biotechnology company spokesperson. Largely missing were experts on legal or ethical issues.

**Table 3:** Categories of spokespeople quoted in Canadian, United Kingdom, and United States newspapers

**Spokesperson**^**a**^	**Percent (%) of articles**			***P*-value**^**b**^
	**Canada (n = 26)**	**United Kingdom (n = 77)**	**United States (n = 55)**	
Researchers from universities/research institutes/government	85	57	38	0.000*
Patient advocacy organizations	27	10	31	0.032*
Affected individuals	19	27	33	0.359
Family of affected individuals	19	11	22	0.259
Friends of affected individuals	4	4	4	1.000
Ethics committees	4	1	0	0.430
Parliament/Congress	4	5	0	0.195
Media/Columnist opinion	8	16	29	0.041*

^a^Categories are not mutually exclusive, therefore add to >100%.

^b^Statistically significant differences (p<0.05) are indicated by *.

The dominant frame in 61% of all stories was a celebration of progress in research with no significant difference in framing across countries. For example, a patient advocate was quoted as saying: “It’s really nothing short of miraculous, .... You’re talking about being able to restore vision in animals that are, essentially, totally blind. This is kind of the first fruit of the revolution in genetic medicine.” [35]. Forty-seven of total articles (30%) were dominantly framed as human-interest stories, and 68% of these depicted the challenges of affected individuals living with genetic retinopathies. Forty percent of human-interest stories portrayed affected patients as heroic or hopeful, while only one article conveyed the challenges of affected individuals through a lens of fatalism, depicting sorrow and hopelessness. The challenges of family members of affected individuals were described in 17% of the human-interest stories, and narratives of heroism and hope were present in 19%. While researchers were frequently quoted in articles, only 4% presented human-interest stories on gene transfer researchers, portraying them as heroes. The remainder of the articles (8%) was presented using a descriptive frame, and displayed a neutral account of the science [16]. Other common frames in science journalism—such as conflict (the dominant frame in coverage of politically charged science topics such as climate change and evolution), economic development, morality, scientific uncertainty, risk, and public accountability [32]—were absent.

### Research design explanations

Explanations of research design lacked detail and context in all countries (Table 4). Most striking was the subtle conflation of research and treatment, for example, “[t]o hear such quick progress in a gene therapy *treatment* is fantastic. We hope this success will lead to more funding of gene therapy *research* into conditions that currently have no cure or treatment” [emphasis added] [36].

**Table 4:** Research design explanations in Canadian, United Kingdom, and United States newspapers

**Research design explanations**	**Percent (%) of articles**			***P*-value**^**a**^
	**Canada (n = 26)**	**United Kingdom (n = 77)**	**United States (n = 55)**	
Gene transfer is research				0.069
Clearly mentioned	23	39	47	
Mentioned, but research/treatment conflation	77	51	45	
Sample sizes stated	42	29	40	0.274
Phase of clinical trial				0.000*
Mentioned	4	3	5	
Not applicable	27	0	0	
Gene mutation causes genetic retinopathies	77	48	71	0.006*
Working copy of mutated gene is transferred to ameliorate disease phenotype				0.000*
Mentioned with accuracy	58	53	21	
Mentioned, but incorrect/misleading terminology (e.g. Gene replacement)	15	3	18	
Viral vector transports the gene of interest				0.078
Explained accurately	35	23	18	
Explained, but viral modification not mentioned	23	8	13	
Working gene is transferred to the back of the eye	65	61	38	0.015*
Gene transfer involves eye surgery	62	56	38	0.063

^a^Statistically significant differences (p<0.05) are indicated by *.

Sixty five percent of articles did not discuss sample sizes in research, and 87% of articles failed to indicate the phases of clinical trials. Canadian articles were more likely to discuss pre-clinical studies (i.e., in vitro or animal studies), while a greater proportion of UK and US articles discussed clinical trials (Table 4). UK articles were half as likely (48%) to state that a gene mutation caused the genetic retinopathy under investigation, compared to Canadian (77%) and US (71%) articles. However, US articles were least accurate in their reporting of the function of gene transfer and the mechanics of its delivery (Table 4). Funding sources were mentioned in only 39% of all articles. Only 3% of articles described controversies and conflicts-of-interest. For example, controversy over the benefits of gene transfer for patients, who had adjusted to their visual impairment was described: “[w]hile the prospect of a cure is exciting, it can also be scary for people living with limited or no sight …. If they said, ‘there’s a cure and you’re going to be able to see’ , they wouldn’t just jump and say, ‘Yes!’ Because the whole world would change on them, … They’ve both been able to carry on with their lives without sight. Then all of a sudden if you could see it wouldn’t make sense … They wouldn’t be able to read” [37].

### Representations of risks, benefits, and timelines

**Figure 2:**
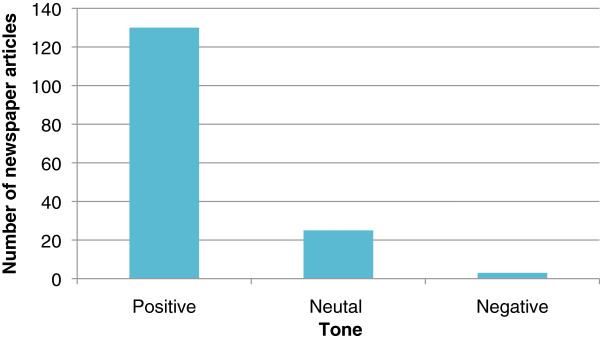
Tone of ocular gene transfer in newspaper articles from Canada, United Kingdom and United States.

**Figure 3:**
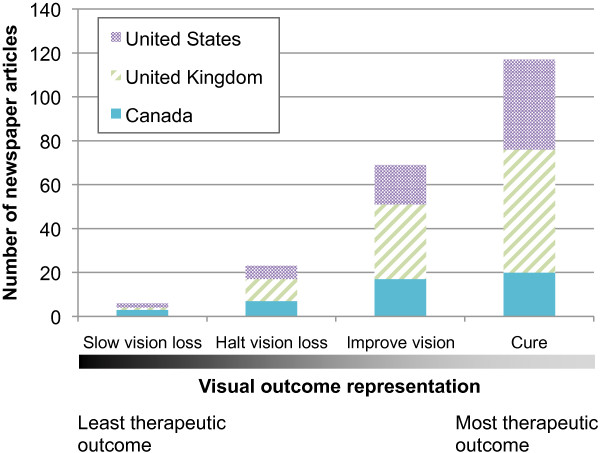
Representations of ocular gene transfer visual outcomes in newspaper articles from Canada, United Kingdom, and United States.

The tone of newspaper coverage was overwhelmingly positive in all countries (Figure 2). Benefits were disproportionately represented with a median of three benefits compared to a median of 0 risks (only 43% of articles described any risk) (Table 5). While some statistical differences existed among countries in reporting of risks, these are due to the very low percentages of articles that discussed specific risks (Table 5). The commonality among countries was the lack of reporting of risks. In general, *no* risks were mentioned in 35% of Canadian, 69% of UK, and 53% of US articles. In contrast, all articles discussed benefits; visual improvement was prominently represented in Canadian (100%), UK (91%), and US (87%) articles. Representations of visual benefit ranged along a continuum of outcomes (Figure 3) from slowing vision loss to a cure. For example, one article claimed, “REVOLUTIONARY gene therapy can cure a severe form of inherited blindness in days, groundbreaking trials show” [38]. Indeed, the more therapeutic the outcome along the continuum, the more frequently it was represented in newspaper articles (Figure 3); slowing down vision loss was rarely discussed compared to complete cure. No article indicated that deriving visual benefit from ocular gene transfer might be unlikely.

**Table 5:** Ocular gene transfer risks and challenges in Canadian, United Kingdom, and United States newspapers

**Gene transfer risks, challenges, or caveats**	**Percent (%) of articles**			***P*-value**^**a**^
	**Canada (n = 26)**	**United Kingdom (n = 77)**	**United States (n = 55)**	
Not mentioned	35	69	53	0.006*
General health risk	19	17	33	0.098
Efficacy concerns	35	9	18	0.003*
New research or first-in-human experimentation	12	8	5	0.607
Long timeline to clinical implementation	15	5	4	0.002*
Historical adverse events in gene transfer clinical trials	8	0	11	0.006*
Eye health risk	8	3	7	0.299
Unknown risk/Uncertain risk	15	0	5	0.003*
Complexity of gene transfer	4	6	0	0.143
Economic risk	11	0	0	0.004*
Ethical challenges	4	0	0	0.075
Social challenges	0	1	0	1.000
Quality of life concerns arising from clinical trial participation	4	0	0	0.165
Legal risk	0	0	0	1.000

^a^Statistically significant differences (p<0.05) are indicated by *.

While the majority of articles predicted visual benefit as an outcome of ocular gene transfer, 87% of all articles did not provide estimates of timelines for the commencement of clinical trials, the phases of clinical trials, the regulatory process, or the clinical implementation of this biotechnology. Table 6 provides examples of statements that predict timelines for the translation of gene transfer research.

**Table 6:** Illustrative timeline estimates for ocular gene transfer clinical trials or clinical application

**Timeline estimate**	**Predicted year for outcome**	**Outcome**	**Source**
“Bennett, an associate professor of ophthalmology at the University of Pennsylvania…said she hopes initial experiments in people can begin within about two years.”	2003	Leber congenital amaurosis clinical trials began in 2007, with phase-I results published in 2008 (Bainbridge et al., [6]; Hauswirth et al., [7]; Maguire et al., [8]).	Anonymous: *Gene therapy restores dogs’ eyesight, may treat blind*. Houston Chronicle; 2001, April 28.
“Within five years [gene transfer] could be ready for testing on people who suffer age-related macular degeneration.”	2013	Phase I clinical trials were initiated in 2010 (NCT01678872).	Highfield R: *Hope of genetic cure for failing eyesight*.
			The Daily Telegraph; 2008, April 28.
“This [phase-I gene transfer clinical trial for Leber congenital amaurosis] really paves the way for developing a treatment for people who have so far had no prospect of a cure,” said Robin Ali, an ophthalmologist at UCL… “Within two to three years it might be approved for use in the clinic.”	2010-2011	Leber congenital amaurosis is currently recruiting for a phase-III clinical trial (NCT00999609).	Sample I: *Maze walk marks ‘huge advance’ in gene therapy for blindness*. The Guardian; 2008, April 28.
“[achromatopsia] treatment could be as little as four or five years away.”	2016-2017	To be determined.	Hilpen K: *The boy who sees in black and white*. The Daily Express; 2012, March 6.

## Discussion

Our analysis of newspaper coverage of ocular gene transfer identifies persistent challenges posed by overly positive representations of experimental biotechnologies in all three countries analysed. Sensationalism in the news media is seldom present as blatantly inaccurate reporting [20,28,31]. Instead, as for ocular gene transfer, overly enthusiastic or exaggerated claims are evident through biased framing, errors of omission, and an emphasis on benefits over risks. Such overly optimistic reporting serves to discredit media claims [39] and build heightened social expectations about the promise of emerging biotechnologies [40]. As such, the research community, policy makers, and ethicists view a lack of balance in media reporting as a major shortcoming [20].

Our analysis of newspaper coverage of ocular gene transfer identified minor differences in coverage in Canadian, UK and US print media, but the same essential concerns persisted. Coverage in all three countries focused on human-interest stories, dominant framing as a “celebration of progress”, and had limited discussion of risks, research design, conflicts-of-interest, funding sources, and research timelines. US news articles were least likely to discuss the genetic cause of retinopathies and were most inaccurate in their depiction of the purpose and methodology of gene transfer. Differences in coverage patterns were likely due to the number of articles in newspapers of varying quality per country (e.g., national versus local and broadsheet versus tabloid) and attention to local research. For example, Canadian articles reported more pre-clinical research, reflecting the state of gene transfer in Canada.

Recommendations to address these media challenges are many and include describing scientific roadblocks and the possibility of findings being invalidated by further studies [30]; encouraging reporters to consult independent sources of information about the implications of scientific advances [41]; and encouraging researchers to disclose conflicts-of-interest to reporters [21,22]. Proposed educational strategies to promote balanced reporting include training researchers to “stick to the facts” when communicating with reporters; training graduate students—as future spokespeople—in effective science communication strategies [20]; inviting reporters to scientific conferences; and creating accessible materials to educate the news media about scientific advances [42].

Unfortunately, these recommendations do not adequately account for the nature of news media, which, as businesses, face their own pressures. Journalists produce copy within limited deadlines and compete fiercely for editorial and public attention. While increasing web content expands potential to provide further background material, print and television media still face space or time constraints. Our findings on frames and emphasis of benefits over risks highlight that ocular gene transfer is seen as a “good news” story for the visually impaired and their families. Our analysis confirms that decades of recommendations and initiatives to improve the tenor and content of science journalism have resulted in limited discernible improvement, at least, in the context of ocular gene transfer. As a result, recommendations may be more tractable at the level of clinical communications between clinicians and patient communities. Here, we first place our results in the context of other studies and then recommend the development of communication strategies between clinicians and their patients, especially potential clinical trial participants, that operate within the landscape of overly optimistic media coverage.

### Frames, benefits, and therapeutic misconception

Researchers from universities and research institutes were the dominant spokespeople, and clinical researchers, in particular, were represented as heroes [43]. The public places a high degree of trust in such researchers [44], and their statements lend credibility to media reports. Ransohoff & Ransohoff characterize the relationship between media and researchers as one of “complicit collaboration” [39], both promoting promise as well as attracting funds and public support [45]. Indeed, researchers who perceive that media attention will promote their work are most often represented in the news media [46]. As such, researchers are compelled by careerist pressures and serve as partisan stakeholders in media communications [13].

Human-interest stories focused on patients and families [47,48], which presented the narrative of gene transfer through a lens of hope, compassion, empowerment, and heroism [49]. Such narratives are a “powerful way of both universalizing and personalizing human experience” [50]. Emotive descriptions generate the most public conversation and reflection [47]; capture the attention of policy makers; and provide a platform for disease advocacy and funding opportunities [48,51]. Nevertheless they may mask the scientific information presented in media reports [47].

While newspaper articles, especially those with longer format, may have more than one frame, in our dataset, the dominant frame was a “celebration of progress”, which was consistent with other novel medical biotechnologies [19,32]. This frame generates the social expectations necessary to sustain public support for gene transfer [40], but detracts from risks and reinforces optimistic messages of benefits [20]. Frames are most effective when they resonate with audiences on a psychological level [52]. As such, frames of progress may reinforce the hopes of patients and their families.

Often missing from the positive frames were explanations of research design [53]—including sample size and study phase—that conflated research with treatment. Conflicts-of-interest or funding sources were similarly omitted. Such omission diminishes transparency and the ability of the public to critically evaluate the stakes of interested and affected parties in the research effort [28,31,53]. Funding or other conflicts-of-interest should always be visible in media reports [21,22], especially in consideration of the checkered history of gene transfer. An early gene transfer trial for ornithine transcarbamylase deficiency at the University of Pennsylvania, which resulted in the death of a young participant, is regarded as “the most famous conflict-of-interest case in medicine” [2]. It resulted in a loss of public trust [5], which is essential for the sustainable translation of this biotechnology [54].

Supporting the positive framing was an overemphasis on benefits at the expense of a discussion of risks [28,31,53,55,56]. Risks are notoriously difficult to explain to lay audiences, including journalists, because of inaccessibility of probabilistic information [57]. Ethical and social risks also go underreported [20,42,58], even though, one study of social media indicated public concern about these [59]. Most importantly, however, serious risks exist for clinical research participants in ocular as well as other gene transfer interventions. Oncogenic risks arise from insertional mutagenesis and a severe immune response to the viral vector has resulted in death [1]. Risks specific to ocular gene transfer include surgical complications, loss of an eye due to inflammation, loss of remaining vision, as well as the very low risk of brain toxicity due to viral vector integration into the optic nerve [6-8,33,60]. A discussion of risks emphasizes the early-stage, experimental nature of the technology, which has historically been associated with risk and uncertainty.

As is common for novel biotechnologies, benefits were the focus of the articles [19,42,55,59]. Media emphasized direct visual benefits, giving patients and families hope for a similar outcome even though most reported studies were pre-clinical or phase I trials, focused on safety not efficacy. Additionally, media articles conflated goals of research and of clinical care: a phenomenon that is termed therapeutic misconception [61]. Therapeutic misonception is ethically problematic because an understanding of the goals of a clinical trial is necessary for autonomous decision-making and informed consent for research participants [62].

The extent to which media coverage contributes to therapeutic misconception is not known. However, one survey of prospective participants for a phase I oncology clinical trial found that exposure to media reports did not result in therapeutic misconception among patients [63]: 47% of patients who first heard about the trial from the news media correctly identified its purpose prior to informed consent compared to only 15% of patients who did not encounter media descriptions. Nevertheless, therapeutic benefit was the most prominent motivator for participation, meaning that therapeutic optimism can coexist with a correct understanding of the purpose of a phase I clinical trial [63].

More concerning is the news media focus on curative discourse—a phenomenon common in media coverage of prospective biotechnologies [49]. With respect to ocular gene transfer, however, the hope for a cure may be misplaced. Jacobson et al. [64] demonstrated that while vision loss can be halted and even improved by restoring function of dormant but otherwise viable photoreceptors, a cure is not theoretically afforded by gene transfer. This is because gene transfer is non-regenerative, and therefore cannot revive degenerated photoreceptors [64]. Recent evidence also suggests that despite visual improvement, retinal degeneration continues to progress in canine models and humans after LCA gene transfer [65], meaning that long-term efficacy may not be established through ocular gene transfer alone.

Media representations of a cure, while catchy, are inaccurate and misrepresent the theoretical promise of gene transfer research. They leave potential clinical trial participants vulnerable to therapeutic misestimation, whereby benefits are overestimated and risks underestimated [62]. While therapeutic misestimation may sometimes be ethically tolerable because an understanding of the exact probability of benefit is not necessary to make an autonomous decision about participation [62], curative perspectives present a misestimation of the magnitude of benefit rather than its probability. While it is impossible to convey exact probabilities for benefits in novel clinical trials, there is an ethical obligation to avoid raising patient hopes for benefits known to be theoretically infeasible.

Finally, the majority of articles failed to contextualize timelines for clinical application of ocular gene transfer. Media articles implied that therapeutic benefits were imminent, despite their early stages of development. Similarly, media portrayals of time estimates for gene discoveries for psychiatric condition and the availability of clinical services were largely unmet [58]. Such portrayals of present research outcomes may be ethically problematic for patients, as they may inflate expectations for a treatment in ongoing or future clinical trials and set patients up for disappointment [23].

In summary, newspaper communications about ocular gene transfer were replete with errors of omission and employed optimistic frames commonly used to generate social expectations about novel biotechnologies [40]. Benefits were over-represented and risks were often not discussed. Moreover, the focus on curative language within a therapeutic spectrum raises challenges for the ethical communication about ocular gene transfer in the context of recruiting clinical trials.

### Limitations

This study only examined newspaper coverage and not Internet or television content, however, leading newspapers have an agenda setting role, serving as a platform for other media venues. With the exception of coding for curative visual benefits, this study did not assess media bias through “errors of commission” [20] as it did not compare the content of media coverage with that of scientific journal and clinical publications. Despite this shortcoming, this study explored the lack of balance in media reporting through the more common forms of omission and framing biases [28,31,32]. Finally, the study did not examine the impact of media coverage directly on potential participants in recruiting clinical trials and their families. While we interviewed patients about the prospect of an ocular gene transfer clinical trial for choroideremia and their responses informed the coding frame, the proposed trial was not yet recruiting participants.

## Conclusions

This study shows little improvement in science media communication in the past decade, despite initiatives to improve journalism on novel health technologies [20,28,30,31,41,42,47]. Media reports continue to be overly optimistic and framed as human-interest stories, whether from the perspective of the heroic researcher or hopeful patient. We therefore reiterate that clinicians and researchers, in their encounters with the news media, should be factual, balanced in addressing both risks and benefits, disclose funding sources and conflicts-of-interest, provide realistic timelines, and discourage curative speculation when research is either in early stages or when a cure is not theoretically possible. As a further incentive for moderated media communications, we support Kimmelman’s [66] recommendation that institutional review boards require investigators of clinical trials to submit a portfolio of their press releases as a component of ethics review [66].

At a more practical level, however, and one that is directly in the control of researchers/clinicians, the onus must be on these stakeholders to address the backdrop of media coverage in the context of ongoing clinical care and clinical trial enrolment. In particular, researchers and clinicians must distinguish between the goals of research, of the phases of clinical investigation, and treatment. Additionally, clinical communicators must counter media messaging, emphasizing to their patients that a cure will not be afforded through gene transfer alone. Taking media communications into account when discussing the potential of ocular gene transfer clinical trials will promote ‘informed hope’ among patients in the context of clinical care [67], informed consent among research participants in the context of recruiting trials, and facilitate the ethical translation of this biotechnology as it moves towards clinical application.

## Abbreviations

LCA: Leber congenital amaurosis.

## Competing interests

The authors declare that they have no competing interests.

## Authors’ contributions

SB and TB conceptualized the research questions and study design. SB collected, coded, supervised coding done by a research assistant, and carried out statistical analyses. SB and TB drafted, edited, and approved the final manuscript.

## Authors’ information

Ms. Shelly Benjaminy has a BSc in molecular genetics and an MSc in health policy from the University of Alberta. Her research explores multi-stakeholder priorities for the translation of novel biotechnologies. Dr. Tania Bubela has a PhD from the University of Sydney in biology and a JD from the University of Alberta. She researches in the field of health law and policy as an Associate Professor, School of Public Health, University of Alberta.

## Pre-publication history

The pre-publication history for this paper can be accessed here:

http://www.biomedcentral.com/1472-6939/15/58/prepub
